# Characteristics of nitrogen deposition research within grassland ecosystems globally and its insight from grassland microbial community changes in China

**DOI:** 10.3389/fpls.2022.947279

**Published:** 2022-08-04

**Authors:** Tong Li, Lizhen Cui, Lilan Liu, Hui Wang, Junfu Dong, Fang Wang, Xiufang Song, Rongxiao Che, Congjia Li, Li Tang, Zhihong Xu, Yanfen Wang, Jianqing Du, Yanbin Hao, Xiaoyong Cui

**Affiliations:** ^1^College of Life Sciences, University of Chinese Academy of Sciences, Beijing, China; ^2^College of Resources and Environment, University of Chinese Academy of Sciences, Beijing, China; ^3^School of Environment and Science, Centre for Planetary Health and Food Security, Griffith University, Brisbane, QLD, Australia; ^4^Chengdu Institute of Biology, Chinese Academy of Sciences, Chengdu, China; ^5^Institute of Ecology and Biodiversity, School of Life Sciences, Shandong University, Qingdao, China; ^6^Institute of Marine Science and Technology, Shandong University, Qingdao, China; ^7^National Science Library, Chinese Academy of Sciences, Beijing, China; ^8^Department of Library, Information and Archives Management, School of Economics and Management, University of Chinese Academy of Sciences, Beijing, China; ^9^Institute of International Rivers and Eco-Security, Yunnan University, Kunming, China; ^10^State Key Laboratory of Tibetan Plateau Earth System Science (LATPES), Institute of Tibetan Plateau Research, Chinese Academy of Sciences, Beijing, China; ^11^Beijing Yanshan Earth Critical Zone National Research Station, University of Chinese Academy of Sciences, Beijing, China

**Keywords:** nitrogen deposition, grassland, keyword co-occurrence, meta-analysis, microbial community

## Abstract

As global change continues to intensify, the mode and rate of nitrogen input from the atmosphere to grassland ecosystems had changed dramatically. Firstly, we conducted a systematic analysis of the literature on the topic of nitrogen deposition impacts over the past 30 years using a bibliometric analysis. A systematic review of the global research status, publication patterns, research hotspots and important literature. We found a large number of publications in the Chinese region, and mainly focuses on the field of microorganisms. Secondly, we used a meta-analysis to focus on microbial changes using the Chinese grassland ecosystem as an example. The results show that the research on nitrogen deposition in grassland ecosystems shows an exponential development trend, and the authors and research institutions of the publications are mainly concentrated in China, North America, and Western Europe. The keyword clustering results showed 11 important themes labeled climate change, elevated CO_2_, species richness and diversity, etc. in these studies. The burst keyword analysis indicated that temperature sensitivity, microbial communities, etc. are the key research directions. The results of the meta-analysis found that nitrogen addition decreased soil microbial diversity, and different ecosystems may respond differently. Treatment time, nitrogen addition rate, external environmental conditions, and pH had major effects on microbial alpha diversity and biomass. The loss of microbial diversity and the reduction of biomass with nitrogen fertilizer addition will alter ecosystem functioning, with dramatic impacts on global climate change. The results of the study will help researchers to further understand the subject and have a deep understanding of research hotspots, which are of great value to future scientific research.

## Introduction

Nitrogen (N) is an essential element for the growth of organisms. Haber-Bosch’s nitrogen conversion project (Erisman et al., 2008) has effectively addresses the growing food demand of billions of humans. However, the unreasonable utilization of N has led to a series of ecological and environmental problems, the most important of which is the rapid increase of atmospheric N deposition. Atmospheric nitrogen deposition is mainly caused by the emission of excess reactive nitrogen compounds from human activities and atmospheric transport processes (Fowler et al., 2013). Grasslands, as the largest terrestrial ecosystem in the world, account for about 30–40% of the total land area (O’Mara, 2012). Grasslands are limited by nutrient availability, due to the relatively poor nutrient background of soils, the relatively low historical N deposition, and nutrient loss induced by grazing and mowing (Chen et al., 2016a). Thus, even chronic N deposition can cause soil eutrophication and acidification (Tian and Niu, 2015). Excess N levels directly affect plant diversity (Payne et al., 2017) and net primary productivity (NPP) (LeBauer and Treseder, 2008), which in turn affect the biogeochemical cycles of C, N, and P (Quinn Thomas et al., 2010; Peñuelas et al., 2013). The profound impact of atmospheric N deposition on grassland ecosystems has generated extensive research interest. Researchers have conducted different types of long-term or short-term experimental simulation and computer model simulations in global grassland ecosystems (Luo et al., 2016), and these studies have become a research hotspot in global change ecology.

**FIGURE 1:**
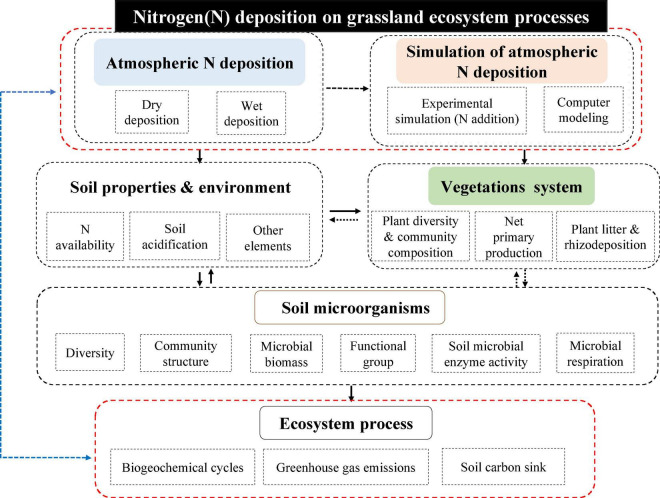
Conceptual diagram of the ecological effects of N deposition in grassland ecosystems. Nitrogen deposition can be divided into atmospheric nitrogen deposition (dry deposition and wet deposition) and artificially simulated nitrogen deposition (artificial addition experiments and computer simulations). The main impacts on grassland ecosystems include soil systems, plant systems, and ecosystem processes, with soil microorganisms in turn playing an important role as drivers affecting the soil-plant-ecological process continuum.

Throughout the history of research on the ecological effects of N deposition (including simulated N deposition, hereafter the same) in grassland ecosystems, relevant research work has focused on the effects of N deposition on the composition, structure, function, and processes of grassland ecosystems. It can be summarized in the following three aspects, as shown in the Figure 1.

(1) Effects of N deposition on the composition, structure and function of vegetation.

Nitrogen deposition significantly alters the species composition of native grasslands because increased the availability of nitrogen in the soil. Enriched nitrogen may be interacting with mycorrhizal feedbacks, allowing a few nitrophilic plant species become dominant while other species to disappear (Johnson et al., 2008). The plants species composition is significantly species-specific in response to nitrogen deposition and is influenced by species properties (Cleland et al., 2006; Shen et al., 2022), environmental factors (e.g., light, temperature, and soil acidity) (Tian and Niu, 2015), and mycorrhizal symbiotic status (Quinn Thomas et al., 2010). Nitrogen deposition can alter ecosystem function and stability by enhancing nitrogen competition between dominant and dependent species, especially in highly dominant grasslands (Liu et al., 2019). Studies have found that changes in species composition led to a decrease in the proportion of forb at high nitrogen addition rates (Bai et al., 2010).

Nitrogen deposition could reduce the plant diversity in grassland ecosystems (Duprè et al., 2010; Payne et al., 2017; Li et al., 2020). Some mechanisms may explain changes in plant diversity, including accumulation pressures with large amounts of N (Payne et al., 2017), mild competition (Ma et al., 2020), soil acidification and its ionic toxicity (Tian et al., 2016), nutrient imbalance (Payne et al., 2017).

Nitrogen deposition significantly reduced the height and coverage of plant (Payne et al., 2013; Wu et al., 2020), richness (Stevens et al., 2004; Bai et al., 2010; Roth et al., 2013), and the Pielou’s index (Tang et al., 2017). Nitrogen deposition could affects ecosystem productivity through its effects on community structure. Nitrogen enrichment could increase plant productivity. When nitrogen deposition lifts the nitrogen restriction of plant growth, plants invest less carbon in the underground part and more carbon in the above-ground part to obtain other restricted resources (e.g., light). Root growth and above-ground net primary productivity (ANPP) accumulation tend to exhibit different response characteristics to nitrogen deposition. For example, Wang et al. (2019) found that in temperate grasslands, at lower N addition levels, ANPP increase with N additions, while below-ground net primary productivity (BNPP) decreases. Nitrogen enrichment could reduce the stability of ANPP on local and larger spatial scales but does not affect the stability of BNPP or NPP at the scale studied (Yang et al., 2022).

(2) Effects of nitrogen deposition on the composition, structure and function of soil systems.

In nitrogen limited ecosystems (e.g., grassland ecosystems in northern China), nitrogen deposition generally relieves the nitrogen limitation of plants and microorganisms (Han et al., 2012). It has been shown that N addition significantly increased the biomass and respiration of plant roots in the nitrogen limited grasslands of Inner Mongolia (Chen et al., 2014, 2016b; Zhang et al., 2022). Nitrogen deposition could directly and indirectly cause a series of changes in soil physicochemical properties (e.g., N effectiveness, soil acidification, base cation composition, etc.) (Zhou et al., 2017) and soil enzyme activity (Henry et al., 2005). Simulated increased nitrogen deposition promoted the activity of soil hydrolytic enzymes (sucrase, cellulase, acid phosphatase, and urease) (Marklein and Houlton, 2012; Jian et al., 2016), inhibited oxidase activity (polyphenol oxidase and peroxidase) (Treseder, 2008; Jian et al., 2016). Nitrogen deposition could directly affect soil microbial biomass, diversity and community structure by changing soil environmental conditions, resulting in a significant decrease in soil microbial respiration (Zhou et al., 2014). It could also indirectly affect the structure of the soil microbial community through the physiological and ecological responses of above-ground vegetation (Chen et al., 2017). In contrast to the consistent response of overall microbial diversity to nitrogen deposition, there are significant differences in the response of different functional groups of microorganisms involved in the N cycle to nitrogen deposition. Key enzyme-related genes encoding N transformation processes, such as the *nifH* gene for N fixation, the *chiA* gene for mineralization, the *amoA* gene for ammonia-oxidizing archaea (AOA) and ammonia-oxidizing bacterial (AOB), and the abundance of *narG*, *nirS*, *nirK* and *nosZ* genes for nitrification is bound to change with increasing N deposition (Chon et al., 2011; Zhang et al., 2013; Zhou et al., 2021).

(3) The influence of N deposition on biogeochemical cycles.

Numerous studies have shown that the accumulation of reactive nitrogen could change the stoichiometric balance of C:N:P in ecosystems. Greenhouse gas (CO_2_, N_2_O, and CH_4_) emissions are ultimately controlled by altering the biogeochemical cycles of carbon, nitrogen and phosphorus (LeBauer and Treseder, 2008; Quinn Thomas et al., 2010). Nitrogen deposition could alleviate nitrogen limitation of soil microorganisms and increase microbial activity and decomposition rate of soil organic matter, which may lead to loss of soil carbon. A global meta-analysis showed that CH_4_ was increased and N_2_O was released due to nitrogen deposition (Liu and Greaver, 2009; Gomez-Casanovas et al., 2016).

In summary, nitrogen deposition not only has a profound effect on soil systems, plant systems, and ecosystem functions and processes, but also has a significant impact on soil microbial communities and presents diverse mechanisms of influence. Some studies have shown that nitrogen deposition has an inhibitory effect on microbial growth and that nitrogen deposition may reduce the activity of plant inter-rooted microorganisms. Different nitrogen deposition classes have different effects on soil microbial load. Microbial biomass carbon (MBC) can reflect subtle changes in the soil environment due to its high sensitivity (Eisenhofer et al., 2019). Therefore, an in-depth understanding of the response of soil microbial biomass to nitrogen deposition in grasslands and its mechanisms is extremely important to maintain the ecological function of grassland ecosystems. Therefore, in addition to a holistic understanding of the response of grassland ecosystems to nitrogen deposition, the importance of microbial biomass response to N deposition and its mechanisms in maintaining the ecological functions of grassland ecosystems should be better understood.

Although a lot of research has been done in the field of grassland nitrogen deposition, there is still a lack of research on the publication situation and quality of in-depth literature in this field. Bibliometric scientific mapping is a quantitative method that analyzes the full range of terms present in scientific publications in their titles, abstracts, and keywords. Meta-analysis is a statistical method used to compare and synthesize the results of studies on the same scientific question. Whether the conclusions are meaningful or not depends on the quality of the included studies. It is often used for quantitative pooled analysis in systematic reviews. The combination of these two analyses allows us to analyze the evolution of grassland nitrogen deposition research and to predict emerging themes within the discipline. Despite the comprehensive and objective nature of the analysis, bibliometric analysis and meta-analysis has been used in many studies within grassland ecosystem, such as remote sensing (Li et al., 2021), soil metagenomics (Vieira et al., 2021), pasture modeling (Nduku et al., 2021). We used bibliometrics to analyze the responses of global nitrogen deposition to grassland ecosystems, and used a meta-analysis method to analyze the effects of nitrogen deposition on soil microbial biomass, taking China’s grassland ecosystems as an example. We focused on the following four scientific questions: (1) What are the publication patterns and status of N deposition in global grassland ecosystems? (2) What are the hot topics of current research? (3) What are the future research trends? (4) Taking grassland ecosystems in China as an example to explore the effects of nitrogen additions on microbial communities? This research could provide a global perspective on the international dynamics of global grassland nitrogen deposition research, and to provide a reference for understanding the interaction and feedback mechanisms between microbial processes and nitrogen deposition in grassland ecosystems from a local perspective.

## Materials and methods

### Data sources and retrieval strategies

The Web of Science database Science Citation Index Expanded (SCI-EXPAND) was used as a data source, with the keywords “grassland” and “N deposition” as search terms. The search time was 1990–2021 by advanced search, and a total of 2021 was retrieved, the total number of publications is 2,786. We performed a bibliometrics analysis based on these literatures. When we further take China’s grassland ecosystem as an example to explore the meta-analysis of the effect of nitrogen addition on soil microorganisms. Therefore, our data is mainly obtained by searching for literature from ISI Web Science, Science Direct, Google, Google Scholar, and CNKI for peer-reviewed journal articles since December 31, 2021. The keywords searched are (nitrogen deposition OR nitrogen addition OR nitrogen enrichment OR nitrogen fertilizer OR nitrogen amendment OR nitrogen elevated) AND (microbial biomass OR microbial communities OR fungi OR bacteria) AND (soil) AND (China), mainly to observe the changes of microbial diversity and biomass related indicators in five major grassland ecosystems in China under nitrogen addition. We have strict standards for the selection of articles, please refer to the Supplementary material for specific standard information.

### Research methodology

Citespace and VOSviewer are commonly used scientific knowledge mapping tools that are important for a systematic and in-depth understanding of a research field. In this paper, we use VOSviewer to explore the basic knowledge, research hotspots, and frontier knowledge in this field, and CiteSpace to analyze keywords burst of research. Keywords can accurately reflect the hot spots of research in the field. In bibliometric analysis, the frequency of keywords is usually used to determine the focus and development trend of a research field, where the size of the circle represents the frequency of keywords; the higher the frequency is, the larger the circle will be (Van Eck and Waltman, 2010). The keywords in this study were cleaned by thesaurus_terms.txt, which comes with VOSviewer software, and visualized using VOSviewer and Paulek64 5.14 software for further analysis and visual debugging. Keyword burst statistics can reflect research hotspots in the field (Chen, 2017). This study used CiteSpace software to map the keyword burst for N deposition.

To further explore the dynamic evolution of N deposition research over time, we introduced a local citation score (LCS) and a global citation score (GCS) to identify the most-cited references at each stage, with higher LCS values implying that the literature is more important to the field of study. In contrast to a literature with a high GCS, this indicates that the literature has attracted the attention of scientists worldwide, including citations from scholars in other fields of study not related to the discipline (Garfield, 2004). Therefore, the LCS is more appropriate for selecting core literature in the field than the GCS, but the GCS value should also be considered, as it reflects the impact of the work beyond the field. In this study, we used HistCite Pro 2.1 software to calculate the LCS and GCS values.

Meta-analysis requires a large amount of data to calculate. Finally, our dataset includes 251 pairs of observations from 95 published literatures. Among them, microbial carbon has 142 observations, bacterial PLFA has 83 observations, fungal PLFA has 87 observations, Shannon index has 99 observations, and Chao1 has 126 observations. Our metadata covers major grassland ecosystems in China, including alpine grasslands, alpine meadows, meadow grasslands, typical grasslands, and desert grasslands. These data cover a wide range of climatic conditions and soil properties for grasslands in northern China. For example, altitude, annual mean temperature, and precipitation are 475–4,745 m, -5.3 to 8.9°C, and160–900 mm, respectively. Soil properties such as pH (4.0–10.0) also showed a wide range. The average nitrogen application rate was 116 kg ha^–1^ yr^–1^ (ranging from 7.5-640 kg ha^–1^ yr^–1^), the average experiment duration was 5.5 years (1–15 years), and the main type of fertilization was the commonly used fertilizer ammonium nitrate (NH_4_NO_3_) and urea.

We chose Hedges’d to calculate effect sizes rather than the natural log response ratio (ln*RR*) because the frequency distribution of bacterial diversity is more unimodal when Hedges’d is used. The Hedges’d is a unitless metric ranging from −∞ to + ∞. The corresponding magnitude and direction of change can be estimated, and this parameter is not affected by small sample sizes. As suggested by Hedges et al. (1999) for meta-analysis, we used a weighted random-effects model because, when dealing with ecological data, when combining data from individual studies, using a random-effects model can reduce the variability of different studies, and when the weights of studies. The larger the value, the higher the weight of these studies, the higher the replication and the lower the variance.

Overall effect sizes were calculated by weighted resampling method from random effects models using MetaWin version 2.0 (Sinauer Associates, Inc., Sunderland, MA, United States). Computational biases were corrected using a bootloader (Dixon, 2020). Missing standard deviations were calculated using the mean coefficient of variation of data sets with known standard deviations according to the method of Geisseler and Scow (2014). If the 95% confidence interval does not overlap with zero at the α = 0.05 level, we consider the effect to be significant at *P* < 0.05 (Hedges and Olkin, 2014). Mineral fertilization type, experimental time, fertilization amount, and ecosystem effects on microbial biomass and diversity were calculated using the procedure described above.

When calculating a random value associated with the between-group (Q_*between*_) statistic, this value describes the heterogeneity of the effects of different groups. The magnitude of the effect is related to the difference in the eigenvalue categories of the research method, based on the chi-square test. In this analysis, grassland ecosystem types are divided into alpine meadow, alpine grassland, meadow grassland, typical grassland, desert grassland; fertilizer types are divided into NH_4_NO_3_ and urea; fertilization time is divided into ≤5, 5–10, >10; application rates ≤50, 50–100, 100–150, 150–200 and >200 kg ha^–1^ yr^–1^. We performed Kendall’s tau rank correlation test (Sokal and Rohlf, 1995) on the data to examine the relationship between the number of replicates per study and standardized effect size (Rosenberg et al., 2000). This relationship suggests publication bias, where larger N effects are more likely to be published than smaller N effects, but we found no associated bias. Analyses were performed using the “metafor” package in R version 4.1.0 (Viechtbauer, 2010). Publication bias for each response variable across the database was assessed using funnel plots. We also detected funnel plot asymmetry by performing Egger’s regression test (Dieleman and Janssens, 2011). All meta-analysis and statistical comparisons were performed using software R 4.1.0.

## Results and discussion

### Analysis of global literature publication characteristics and bibliometric analysis

#### Annual publication trend

**FIGURE 2:**
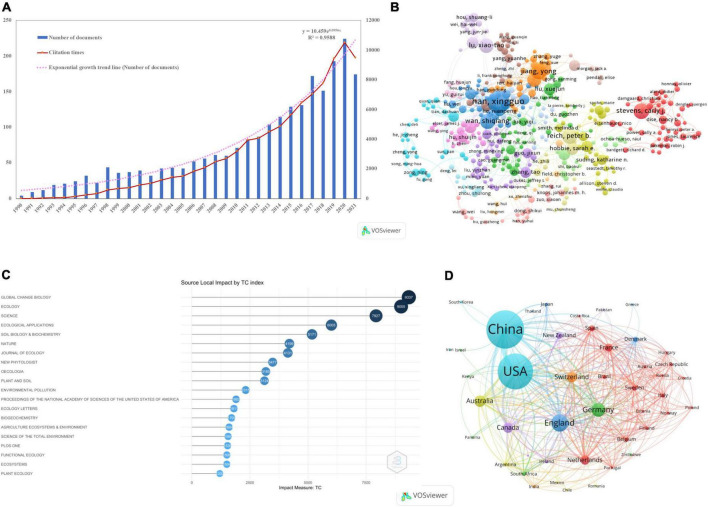
Temporal trend and change point of all papers and cumulative papers in terms of annual publications **(A)**; the network visualization map of 598 authors out of 8,173 authors with more than five publications as produced by VOSviewer **(B)**; top 20 journals based on total local citations as produced by R **(C)**; the network visualization map of 43 countries out of 89 countries with more than five publications as produced by VOSviewer **(D)**.

A total of 2,815 papers were obtained after cleaning the subject dataset and eliminating irrelevant papers. The distribution of publications on N deposition in global grassland ecosystems from 1990 to 2021 (Figure 2A). The results show that research on N deposition in grassland ecosystems shows an “exponential growth” model, indicating that N deposition is developing rapidly in grassland ecosystem research. Based on the changes in the annual literature volume, the research on N deposition in grassland ecosystems can be divided into three stages: (1) the nascent period (1990–1997), where the number of publications increased steadily with time, and the average annual number of publications was less than 20, indicating that the topic of N deposition in grassland ecosystems did not attract extensive attention from scholars in the early stage; (2) the basic exploration period (1998–2011), the number of articles published increased rapidly, but the average annual number of articles published was less than 60; and (3) The number of articles published in the period of rapid development (2012–2021) exceeded 100 and showed a significant upward trend overall, indicating that this research area has stimulated the interest of scientists in recent years and that N deposition in grassland ecosystems has become a hot research area in academia.

Supplementary Table 1 lists the five most cited references that have constructed the knowledge base for N deposition research over three consecutive periods beginning in 1990. These core publications will be identified and listed according to their LCS values. During the nascent period (1990–1996), we identified the five core references with the highest LCS values, which included Tilman (1993), Morecroft et al. (1994), Wilson et al. (1995), Vitousek et al. (1997) reviewed the negative impacts of accelerated input rates and alterations in the terrestrial ecological N cycle from human activities leading to increased N_2_O, a potent greenhouse gas, loss of soil nutrients, potassium and calcium, soil acidification and loss of biodiversity, with the highest LCS values at this stage (268), and Mountford et al. (1993), Tilman (1993), Morecroft et al. (1994) and others found a decrease in species diversity through the application of N treatments by modeling N deposition, and these studies suggest that early on they attracted the attention of scholars in the field of N deposition research. During the fermentation phase (1998–2011), Stevens et al. (2004) had the highest LCS values (380), followed by Clark and Tilman (2008), LeBauer and Treseder (2008), and Bai et al. (2010), and these studies inspired the field of this study during this phase, which focused on N deposition studies on species richness (Stevens et al., 2004; Clark and Tilman, 2008; LeBauer and Treseder, 2008; Bai et al., 2010). In the developmental “leapfrog” phase (2012–2021), Phoenix et al. (2012), Isbell et al. (2013), and Borer et al. (2014) were the three highest LCSs in this phase, followed by Wei et al. (2013) and Yang et al. (2012). The N deposition studies at this stage did not only focus on the impact of N deposition on biodiversity (Phoenix et al., 2012; Isbell et al., 2013; Borer et al., 2014), and there are many studies concerned with the effects of N deposition on plant–soil–microbial systems (Wei et al., 2013). Notably, Stevens CJ’s work is the most cited of the N deposition studies for the entire period (1990-2021), and his findings show that long-term N deposition significantly reduces plant species richness and that species richness decreases linearly with the rate of inorganic N deposition (Stevens et al., 2004).

#### Author analysis

A total of 9,384 authors were involved in the study of N deposition in grassland ecosystems (Figure 2B). A total of 434 authors from 18 clusters published at least five articles on N deposition in grassland ecosystems (Figure 2B and Supplementary Table 2), and the top ten most published authors were Han XG (95), Lu XT (61), Reich PB (53), Stevens CJ (50), Wan SQ (47), Jiang Y (38), Hobbie SE (36), Wang RZ (36), Xu ZW (34), and Tilman D (31). The TLS (total link strength) calculated by VOSviewer reflects the extent of close collaboration between authors. Han XG’s TLS value was 86, which was twice that of Jiang Y (37), indicating that Han XG had more cooperative work. His most representative work was trade-offs and thresholds for modeling the effects of N deposition on grassland biodiversity and ecosystem function in Inner Mongolia (Bai et al., 2010), which has been cited >500 times.

#### Countries and institutes’ analysis

There were 43 out of 89 countries that published a minimum of five publications on N deposition in grassland ecosystems (Figure 2D). China was the most productive country, with 1,053 articles, 5,025 citations, and 4.8 citations per article. The second was the United States, with 968 articles, 7,764 citations, and 8.0 citations per article. The United Kingdom ranked third, with 338 articles, 3,307 citations, and 9.8 citations per article, while Germany and Australia ranked fourth and fifth, respectively (Supplementary Table 2). The TLS (total link strength) calculated by VOSviewer reflects the degree of close cooperation between countries. The United States ranks first with a TLS value of 809, followed by China with 669, indicating that although China has the largest number of publications, it is lower than the United States in terms of international cooperation. China is working more closely with the United States, Canada, the United Kingdom, Germany, and France (Figure 2D). A total of 2,173 institutions worldwide were found to contribute to N deposition in grassland ecosystems (Supplementary Table 2). The top 10 research institutions ranked according to a total national publication, a volume published a total of 1,548 articles, with Chinese research institutions, accounting for 70%, with the Chinese Academy of Sciences (CAS) (660) and the University of Chinese Academy of Sciences (UCAS) (275) leading the way, followed by the University of Minnesota System (134). The CAS is ranked first in terms of TLCS at 4,140, followed by the University of Minnesota System with a TLCS value of 2,027, while Germany and Australia, the top five countries in terms of total publications, do not have institutions in the top 10 list.

#### Influential journals

The 440 journals included in SCIE, the top 20 (4.54%) journals in terms of productivity published a total of 1,211 papers, representing 43.81% of the total literature, with *Plant and Soil* (127, 4.59%), *Global Change Biology* (124, 4.49%), and *Soil Biology & Biochemistry* (112, 4.05%) being the three most published journals; however, 201 journals (45.68%) published only one paper, and 382 journals (86.81%) published fewer than 10 papers (Figure 2C and Supplementary Table 3). *Global Change Biology* ranked first with 9,337 total citations, followed by *Ecology* with a total of 9,005 citations, but the *Ecology* journal ranked first in terms of average citations per paper (120.07), confirming that research on N deposition in grassland ecosystems is focused on global change and ecological issues (Sala et al., 2000; Yang et al., 2019). The impact factors of the journals *Global Change Biology* (10.86) and *New Phytologist* (10.15) are greater than 10, which means that the papers published in these two journals have the highest academic value and are the most referred to by the academic community. Therefore, the three journals *Global Change Biology*, *Ecology*, and *New Phytologist* should be focused on and tracked in the study of N deposition in grassland ecosystems.

### Co-occurrence keyword and burst keyword analysis of research hotspots and trend analysis

#### Co-occurrence keyword analysis

**FIGURE 3:**
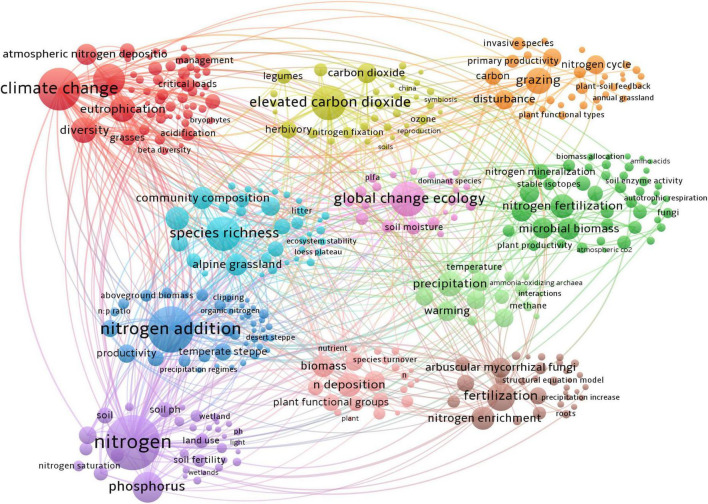
Network of author keywords based on the co-occurrence method on N deposition research from 1990 to 2021.

A total of 389 keywords co-occurrence map (Figure 3) was obtained from 6,201 keywords, with each color cluster on the map reflecting a different research theme in the field of N deposition. The results show that N deposition research could be divided into 11 clusters within different colors, with the red section focusing on climate change and biodiversity. The yellow section represents studies of increasing CO_2_ concentrations. The orange section represents studies of grazing disturbances in the N cycle. The green section focuses on the effects of N fertilizer application on soil microbial biomass, C and N nutrient cycling, and soil physicochemical properties. The fuchsia section is mainly related to global ecosystem change. The light blue section mainly represents studies of species richness and diversity in places such as alpine grasslands and the Tibetan Plateau. The blue section is mainly related to studies of N application on productivity and econmetrics in temperate grasslands, semiarid grasslands, and Inner Mongolia. The purple section focuses on the study of N and phosphorus nutrients. The pink section mainly represents studies of N deposition on biomass, plant functional groups, and species regeneration. The coffee color mainly represents the effect of fertilizer application on N enrichment and the clumping of mycorrhizal fungi. The light green color is mainly related to precipitation, N_2_O, and methane, the main greenhouse gas emissions. Details of the top 10 keywords for each cluster are shown in Table 1.

**TABLE 1:** Identified clusters of author keywords on N deposition research from 1990 to 2021.

ID	color	Cluster name	L	TLS	W	Top 10 author keywords
#1	Red	Climate change	198	438	157	Biodiversity, eutrophication, diversity, atmospheric, N, deposition, grasses, critical loads, species composition, land-use change, acidification, management
#2	Yellow	Elevated carbon dioxide	131	292	111	Carbon dioxide, herbivory, legumes, photosynthesis, N fixation, soil N, ozone, CO_2_ enrichment, respiration
#3	Orange	Grazing	125	181	68	Disturbance, N cycle, nutrient limitation, carbon, invasive species, primary productivity, restoration, invasion, plant functional types, water
#4	Green	N fertilization	101	155	60	Microbial biomass, soil respiration, carbon sequestration, carbon cycle, nitrate, N mineralization, soil organic carbon, ^15^N tracer, soil organic matter, ammonium
#5	Red Purple	Global change ecology	166	328	109	Soil moisture, plant community, fire, microbial community, soil properties, ecosystem functioning, PLFA, precipitation change, coastal sage scrub, lignin
#6	Light blue	Species richness	135	340	110	Alpine meadows, community composition, alpine grassland, Tibetan plateau, species diversity, Qinghai-Tibetan plateau, functional groups, community structure, functional traits, nutrient addition
#7	Blue	N addition	179	420	176	Productivity, ecological stoichiometry, temperate steppe, nutrient enrichment, semiarid grassland, above-ground biomass, soil microbial biomass, steppe, temperate grassland, stoichiometry
#8	Purple	Nitrogen	225	709	239	Phosphorus, nutrients soil, soil pH, decomposition, land use, pasture, soil carbon, soil fertility, biogeochemistry
#9	Pink	N deposition	92	148	58	Biomass, competition, drought, plant functional groups, litter decomposition, nutrient cycling, soil microbial community, litter quality, plant-soil (below-ground) interactions, N
#10	Brown	Fertilization	133	256	80	Nitrogen enrichment, arbuscular mycorrhizal fungi, plant diversity, soil acidification, tallgrass prairie, mowing, global warming, extracellular enzymes, nutrient availability, structural equation model
#11	Light green	Precipitation	115	221	64	Warming, greenhouse gas emissions, meta-analysis, nitrification, nitrous oxide, denitrification, temperature, climate warming, N_2_O emission, methane

#### Burst keyword analysis

**FIGURE 4:**
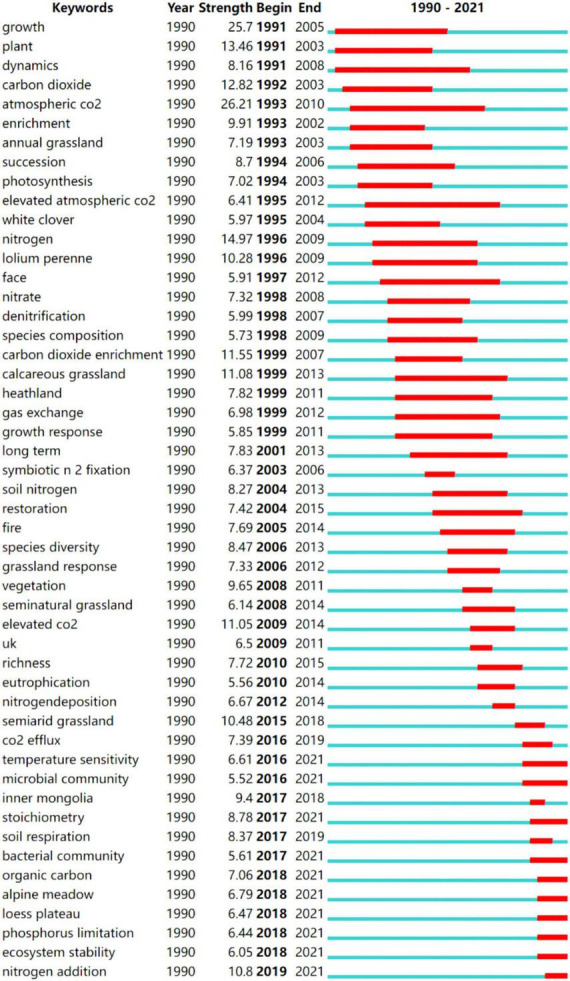
Top 50 keywords with the strongest citation bursts (the red bars indicate some keywords cited frequently; the green bars indicate keywords cited infrequently).

The analysis of the development path and publication time of the keywords indicates a gradual evolution of N deposition research (Figure 4). From 1990 to 2000, the grasslands that were the focus of N deposition research were calcareous grassland and Heathland (Johnson et al., 1998; Lee and Caporn, 1998), and the types of plants of interest was *Lolium Perenne* (Bardgett et al., 1999). The indicators of interest were plant growth, photosynthesis and gas exchange (Morecroft et al., 1994; Van Der Heijden et al., 2000), as well as soil C and N cycle dynamics and root system dynamics (Thornley et al., 1995; Norby and Jackson, 2000), were in response to N deposition, with a particular focus on grassland ecosystems in the context of atmospheric CO_2_ (burst value of 26.21) and N deposition (Lloyd, 1999).

From 2000 to 2010, there was a continued focus on the impact of N deposition on CO_2_, which increases net soil CO_2_ emissions and offsets carbon sequestration by vegetation and soil fractions (Xu et al., 2004). Jiang et al. (2010) showed that N deposition tended to reduce CO_2_ emissions and CH_4_ uptake and increase N_2_O emissions. Attention was also paid to the effects of N deposition on the biodiversity and richness of grassland ecosystems. Stevens et al. (2004) found that long-term N deposition significantly reduced plant species richness through a study of 68 acid grasslands in the United Kingdom and that the rate of this decline was linearly related to the rate of inorganic N deposition. The main cause is the accumulation of N, which increases the competitiveness between species and creates unfavorable soil conditions unfavorable for species growth (Bobbink et al., 2010), such as consumption of soil cations leads to soil acidification, metal migration, and eutrophication (Van Landuyt et al., 2008), which results in changes in flora, and in addition, increases in soil N levels caused by atmospheric N deposition or other means may increase the dominance of annuals and may promote the invasion of new species, which may reduce the diversity of native annuals, while increases in the biomass of exotic annual grasses may also increase the frequency of fire (Brooks, 2003).

From 2010 to 2021, scientists continued to focus on the study of N deposition on CO_2_ fluxes in grassland ecosystems. Long-term atmospheric N deposition promotes soil CO_2_ emissions from alpine meadows on the Qinghai-Tibet Plateau by increasing the soil fast-acting N content and promoting plant growth (Fang et al., 2012). By meta-analysis, Deng et al. (2020) showed that soil organic carbon content and CO_2_ emission fluxes increased by 3.7 and 0.3%, respectively, under N deposition at the global scale and that grassland ecosystems are important greenhouse gas sinks. The results indicate that researchers in this period were more concerned with the study of the effects of N deposition on the physicochemical properties (soil stoichiometric characteristics, soil respiration, soil organic carbon, P limitation) of grassland soils.

Since 2017, the keywords Inner Mongolia, alpine meadow, Loess Plateau, and other locations have become the dominant bursts with burst values of 9.4, 7.06, and 6.79. The research areas of N deposition in grassland ecosystems at this stage are mainly concentrated in Inner Mongolia temperate grassland (Tian et al., 2016), semiarid grasslands (Wang et al., 2019), alpine grassland (Jiang et al., 2018), and the Loess Plateau (Chen et al., 2018) in China. The effect of nitrogen deposition on microbial is an important hotspot in current academic research (Zhang et al., 2013; Zhao et al., 2021; Wang et al., 2022).

### Effects of N addition on microbial biomass and alpha diversity in grassland ecosystems—A case study of Chinese grassland ecosystems

#### Effect of nitrogen addition on soil microbial growth

**FIGURE 5:**
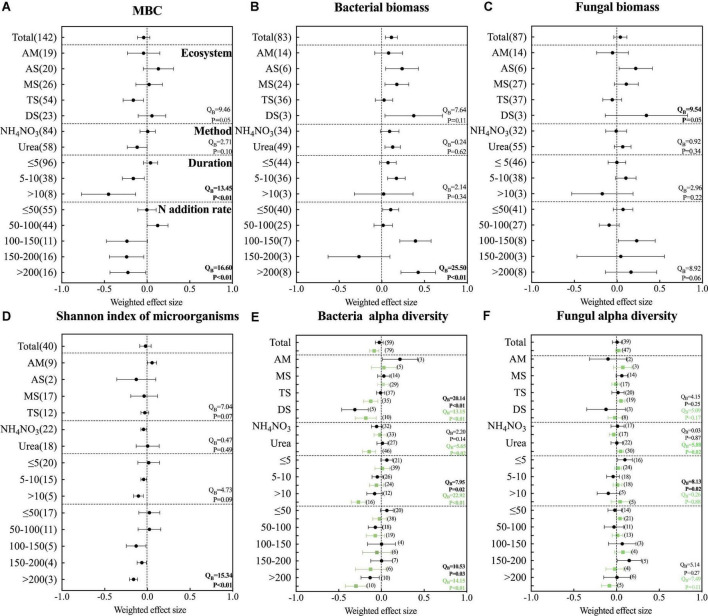
Effects of N addition on **(A)** microbial carbon, **(B)** bacterial biomass, **(C)** fungal biomass, **(D)** shannon index of microorganisms, **(E)** bacterial alpha diversity and **(F)** fungal alpha diversity. The variables are categorized into different ecosystems (AM, alpine meadow; AS, alpine steppe; MS, meadow steppe; TS, typical steppe; DS, desert steppe); fertilizer types (NH_4_NO_3_ and urea), experiment durations, and N application rates. Error bars present 95% confidence intervals. The dashed line was drawn at a mean response ratio = 0. If 95% CI does not overlap zero, then microbial diversity in fertilized soils differed significantly from those in unfertilized soils. The numbers in parentheses next to the point represent the number of studies. In Panels **(E,F)**, black represents Shannon index and green represents chao1 index.

The forest plot showed (Figure 5) that in the grassland ecosystem, the biomass of bacteria increased significantly under nitrogen addition, while the biomass of fungi did not change significantly. Simultaneous nitrogen addition did not alter soil microbial carbon (MBC).

Among them, we found that different grassland ecosystem types responded differently to nitrogen addition. Nitrogen addition significantly increased bacterial biomass in alpine steppe, meadow steppe, and desert steppe ecosystems, while nitrogen addition had no significant effect on bacterial biomass in alpine meadow and typical steppe. Fungal biomass increased only with nitrogen fertilizer addition in alpine steppe ecosystems. Meanwhile, microbial carbon only decreased with nitrogen addition in typical grassland ecosystems and was not significant in other ecosystems. The bacterial biomass is more sensitive to urea, and the addition of urea could significantly increase the bacterial biomass. Fungi are not affected by the type of fertilization. With the increase of fertilization years, the microbial carbon decreased significantly. Bacterial biomass increased significantly at fertilization time 5–10 years. When the fertilization time is more than 10 years, the results here are not significant, probably because of less research. Overall, with the increase of fertilization years, the bacterial biomass increased significantly. However, the biomass of fungi did not change much with the increase of fertilization time. The biomass of bacteria increased significantly with the increase of fertilization rate, while the microbial carbon decreased significantly with the increase of nitrogen application rate.

These results represent that in nitrogen-limited grassland ecosystems, nitrogen addition relieves microbial nitrogen limitation, providing evidence that nitrogen addition can increase bacterial biomass in grassland ecosystems. It was also found that bacteria were more sensitive to nitrogen addition than fungi.

#### Effect of nitrogen addition on soil microbial alpha diversity

In all studies, the effect of nitrogen addition on the Shannon index of soil microorganisms was not significant. Nitrogen addition only significantly decreased the Chao 1 index of bacteria. Different grassland ecosystem types had no significant effect on the total microbial diversity and fungal diversity. Changes in bacterial diversity respond inconsistently across ecosystem types. In a typical ecosystem, the Chao1 index of bacteria decreased significantly with nitrogen addition, but the Shannon index did not change significantly. In the desert-grassland ecosystem, both the Shannon index and Chao 1 index of bacteria decreased significantly. The addition of urea significantly decreased the Chao 1 index of bacteria and significantly increased the Chao 1 index of fungi. The microbial diversity and bacterial diversity decreased gradually with the increase of fertilization time. The diversity of microorganisms and bacteria also decreased significantly with the increase of fertilization rate. Fungal diversity was not significantly affected by the time and nitrogen fertilizer addition rate.

Therefore, our results show that the addition of nitrogen fertilizer could significantly reduce the microbial diversity and bacterial diversity to a certain extent. Fungal diversity was not significant for nitrogen addition.

#### Multivariate relationships between the responses of soil microbes and nitrogen addition

**FIGURE 6:**
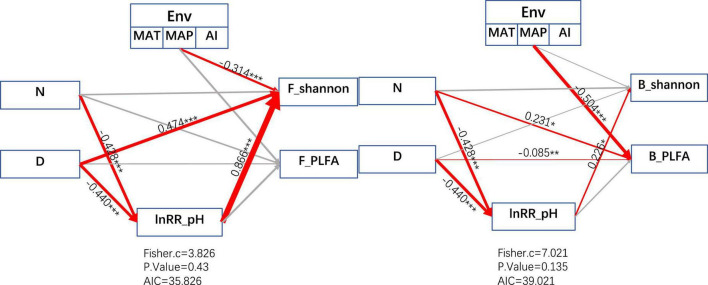
Structural equation modeling (SEM) depicting the effects of multiple drivers on bacterial (B) and fungal (F) Shannon index response ratios and PLFA. D: duration of N fertilizer addition experimental time (years); N: fertilizer addition amount (kg ha^–1^ yr^–1^); ln*RR*_pH is the response ratio of soil pH to microbial PLFA and Shannon diversity under N addition. Environmental factors (ENV) include annual mean temperature (MAT), annual rainfall (MAP), and aridity index (AI). Red arrows and gray arrows indicate significant or insignificant correlations, respectively. The numbers adjacent to the arrows are the normalized path coefficients, which are like relative regression weights and represent the magnitude of the effect of the relationship. The thickness of the arrows is proportional to the magnitude of the normalized path coefficients or covariance coefficients. Fisher.C: Fisher’s C statistic; *P* Value: probability level; AIC: Akaike’s information criterion.

According to the obtained structural equation model (Figure 6), it can be obtained that changes in the environment will reduce the alpha diversity of fungi (*r* = −0.314) and will reduce the biomass of bacteria (*r* = −0.504). And the duration of nitrogen addition and the amount of fertilizer addition will change the alpha diversity of bacteria and fungi by reducing the pH of the soil environment. The Shannon index of fungi will be increasesd by an increase in fertilization time (*r* = −0.474). The time of fertilization directly reduces the biomass of bacteria (*r* = −0.085), and the amount of fertilization positively affects the biomass of bacteria (*r* = 0.231). The Shannon index of bacteria was positively influenced by the ln*RR* of pH indirectly through the regulation of fertilization time and fertilization amount (*r* = 0.226).

Our structural equation model showed that for nitrogen-limited grassland ecosystems, the addition of nitrogen fertilizers could alleviate the nitrogen limitation of microorganisms to a certain extent and increase the diversity and biomass of microorganisms. At the same time, it was also proved that the reduction of soil pH with nitrogen fertilizer addition can positively affect the alpha diversity of microorganisms.

## Conclusion

Publications on N deposition in grassland ecosystems were analyzed by bibliometric analysis from 1990 to 2021 based on data from SCI-EXPAND. The results show that (1) The topic of “grassland” and “N deposition” studies have grown exponentially (*R*^2^ = 0.9588), and the publication trend can be divided into three periods. Before 1997, there was slow, but steady progress (<20 publications per year) in the field. But thereafter, with over 100 publications, and the average annual number increased by more than 5-fold from 2012 to 2021. (2) Research teams in China and United States make the greatest contributions and have a relatively high influence on this research field. Among them, the CAS, the UCAS, and the University of Minnesota System have performed prominently in the number of literature and citations in recent years. (3) The journals with the highest academic value articles by researchers of “N deposition in grassland ecosystems” are global change biology, ecology, and new phytologist. (4) The co-occurrence keyword analysis shows that “N deposition in grassland ecosystems” studies can be categorized into 11 main research themes, including climate change, elevated CO_2_, grazing, species richness, and diversity, etc. (5) Moreover, based on burst keyword analysis, we identified mainly three potential or not fully explored topics: CO_2_ efflux, fragile grassland ecosystems (Inner Mongolia temperate grassland, semiarid grasslands, alpine grassland, and the Loess Plateau), and microbial community. Our further meta-analysis results showed that the addition of N changed the living environment of microorganisms by changing the pH value of the soil, resulting in the rapid growth of acidophilic microorganisms, and the rapid reduction of some microorganisms that were not suitable for the acidic environment, resulting in a decrease in biomass. The diversity of microorganisms will change. Due to the action of hyphae and mycorrhizal fungi, they can perform good self-regulation, so the biomass and diversity of fungi are not as obvious as those of bacteria. This comprehensive study can provide a preliminary analysis of the progress and development trend of N deposition research on global grassland ecosystems. Meanwhile, meta-analysis as a specific case study can further understand the process and mechanism of N deposition impact on grassland microorganisms, which provides an important and important reference for the overall improvement of basic research and ecological risk management of N deposition ecological effects, as well as the grasp of future hotspot areas and key microbial processes. In summary, this research could provide a global perspective on the international dynamics of global grassland N deposition research, and to provide a reference for understanding the interaction and feedback mechanisms between microbial processes and N deposition in grassland ecosystems from a local perspective.

## Data availability statement

The original contributions presented in this study are included in the article/Supplementary material, further inquiries can be directed to the corresponding author.

## Ethics statement

This study was reviewed and approved by the University of Chines Academy of Sciences of Ethics Committee.

## Author contributions

TL, ZX, YW, and XC: conceptualization. TL, LC, and LL: data curation, investigation, and visualization. XC and YW: funding acquisition. TL, ZX, XS, and RC: methodology. HW and YW: resources. CL: software. ZX, YW, and XC: supervision. TL, YW, and XC: validation. TL: writing—original draft. TL, LC, LL, JQD, HW, YH, XC, LT, CL, FW, ZX, and XC: writing—review and editing. HW, JFD, and XC: academic editing. All authors have read and agreed to the published version of the manuscript.
