# Supplementary material for: Characteristics of nitrogen deposition research within grassland ecosystems globally and its insight from grassland microbial community changes in China
Source: Front Plant Sci. 2022 Aug 4;13:947279. doi: 10.3389/fpls.2022.947279 (PMC9386444; doi:10.3389/fpls.2022.947279)
Supplement: Supplementary file 1 [file Data_Sheet_1.PDF]

Supplementary Material

1 Data collection

To explore the effects of simulated nitrogen addition on soil microbial diversity and biomass, we used a meta-analysis the results of published data (Hedges et al., 1999). We searched online databases (ISI Web Science, Science Direct, Google, Google Scholar, and CNKI) for peer-reviewed journal articles since 2021.12.31. The keywords searched are (nitrogen deposition OR nitrogen addition OR nitrogen enrichment OR nitrogen fertilizer OR nitrogen amendment OR nitrogen elevated) AND (microbial biomass OR microbial communities OR fungi OR bacteria) AND (soil) AND (China). In the research to incorporate data from the literature into the database, the following selection criteria must be met:

(1) In the collected studies, the determined mean, standard deviation (SD), and number of replicates must be included. If a suitable article lacks corresponding data, we will contact the author to obtain the original data for research. If the article is displayed in the form of pictures, we will use WebplotDigitizer-4.2 Software (http://digitizer.sourceforge.net) for data extraction. For many studies, the error bar on the histogram is the standard error, and the standard error (SE) must be further converted into the SD according to the transformation. When extracting data from the picture, it is necessary to carry out reasonable conversion and statistics of the data according to the relevant introduction of the article.

(2) The control and treatment of the study are located in the same place. In this way, it can be determined that the differences observed in each study can be attributed to the effects of nitrogen addition and not to spatial soil heterogeneity.

(3) Only choose long-term experiments (>1 year) to remove the microbial and soil physical and chemical changes caused by the excitation effect of soil caused by nitrogen addition.

(4) Nitrogen addition must be included in the article. The addition of nitrogen only includes inorganic nitrogen such as ammonium nitrate and urea. The research needs to include an indicator of microbial biomass or microbial diversity.

(5) In order to focus only on the response of soil microorganisms, soil physics and chemistry, and plants to nitrogen addition, avoid collecting comprehensive experiments that add organic nitrogen or the coupling of nitrogen fertilizer and other elements. Most of this type of research focuses on agricultural research rather than exploring the consequences of nitrogen deposition.

(6) If the article contains multiple sampling times and multiple levels of soil depth, some and only consider the latest sampling time and the microbial changes in the topmost soil.

For each study in the database, we recorded experimental location (longitude and latitude), climatic factors (annual mean temperature and precipitation, MAT and MAP), grassland ecosystem type, nitrogen fertilizer type, experimental duration (years), nitrogen application rate (kg ha^{-1} yr^{-1}), microbial biomass (bacterial PLFA, fungal PLFA, MBC), microbial diversity indicators (Shannon or Chao1). If there are other soil physical and chemical properties in the article, including soil organic carbon, soil total nitrogen, C/N ratio, and pH value, record them to make the data as complete as possible. Meanwhile, the annual mean temperature (°C) and the annual mean precipitation (mm yr^{-1}) were directly extracted from the cited papers, and if not reported, ArcGIS (Version 10.0, ESRI, Redlands, CA) was used according to Location information (i.e., latitude and longitude) is extracted from the WorldClim database (http://www.worldclim.com).

Finally our dataset includes 251 pairs of observations from 95 published literatures. Among them, microbial carbon has 142 observations, bacterial PLFA has 83 observations, fungal PLFA has 87 observations, Shannon index has 99 observations, and Chao1 has 126 observations. Our metadata covers major grassland ecosystems in China, including alpine grasslands, alpine meadows, meadow grasslands, typical grasslands, and desert grasslands. These data cover a wide range of climatic conditions and soil properties for grasslands in northern China. For example, altitude, annual mean temperature and precipitation are 475 to 4,745 m, -5.3 to 8.9°C and160 to 900 mm, respectively. Soil properties such as pH (4.0-10.0) also showed a wide range. The average nitrogen application rate was 116 kg ha^{-1} yr^{-1} (ranging from 7.5-640 kg ha^{-1} yr^{-1}), the average experiment duration was 5.5 years (1-15 years), and the main type of fertilization was the commonly used fertilizer ammonium nitrate (NH_4NO_3) and urea.

2 Meta-analysis:

We assessed the effect of mineral fertilization on soil microbial biomass and diversity using Hedges'd, which represents effect size. We chose Hedges' d to calculate effect sizes rather than the natural log response ratio (lnRR) because the frequency distribution of bacterial diversity is more unimodal when Hedges' d is used. The Hedges'd is a unitless metric ranging from $-\infty$ to $+\infty$. The corresponding magnitude and direction of change can be estimated, and this parameter is not affected by small sample sizes. Calculated using the following equation (Hedges & Olkin, 2014):

$$d = \frac{(X_e - X_c)}{S} J$$

Where d is the magnitude of the effect on soil microbial biomass and diversity, and X_c and X_e represent the corresponding values of the selected microbial variables in the unfertilized control and fertilizer treatments, respectively. The S and J terms are the

pooled standard deviation and correction factor, calculated from the following equations:

$$S = \sqrt{\frac{(N_e - 1)S_e^2 + (N_c - 1)S_C^2}{N_c + N_e - 2}}$$

$$J = 1 - \frac{3}{4(N_c + N_e - 2) - 1}$$

Where S_c and S_e represent the standard deviation of microbial variables in the unfertilized (control) and fertilized treatments. The terms N_c and N_e denote the sample size of microbial variables in the unfertilized and fertilized treatments.

Overall effect sizes were calculated by weighted resampling method from random effects models using MetaWin version 2.0 (Sinauer Associates, Inc., Sunderland, MA, USA). Computational biases were corrected using a bootloader (Dixon, 2020). Missing standard deviations were calculated using the mean coefficient of variation of data sets with known standard deviations according to the method of Geisseler and Scow (2014). If the 95% confidence interval does not overlap with zero at the $\alpha = 0.05$ level, we consider the effect to be significant at $P<0.05$ (Hedges & Olkin, 2014). Mineral fertilization fertilization type, experimental time, fertilization amount, and ecosystem effects on microbial biomass and alpha diversity were calculated using the procedure described above.

To determine the effects of grassland ecosystem type, fertilizer type, fertilization time, and fertilization amount on soil microbial biomass and diversity, a random-effects model was used to calculate the overall effect size heterogeneity between groups. Each attribute was divided into categories to determine whether there were significant differences in effect size between categories. When calculating a random value associated with the between-group ($Q_{between}$) statistic, this value describes the heterogeneity of the effects of different groups. The magnitude of the effect is related to the difference in the eigenvalue categories of the research method, based on the chi-square test. In this analysis, grassland ecosystem types are divided into alpine meadow, alpine grassland, meadow grassland, typical grassland, desert grassland; fertilizer types are divided into NH_4NO_3 and urea; fertilization time is divided into ≤5, 5-10, >10 ; application rates ⩽50, 50–100, 100-150, 150-200 and >200 kg ha^{-1} yr^{-1}. We performed Kendall's tau rank correlation test (Sokal & Rohlf 1995) on the data to examine the relationship between the number of replicates per study and standardized effect size (Rosenberg et al., 2000). This relationship suggests publication bias, where larger N effects are more likely to be published than smaller N effects, but we found no associated bias. Analyses were performed using the "metafor" package in R version 4.1.0 (Viechtbauer et al., 2010). Publication bias for each response variable across the database was assessed using funnel plots. We also detected funnel plot asymmetry by

performing Egger's regression test (Dieleman and Janssens, 2011). All meta-analysis and statistical comparisons were performed using software R 4.1.0.

3 Structural equation modeling

To assess the relationship between lnRR of soil microbial biomass and diversity and nitrogen application rates, timing, and environmental variables. We employed structural equation modeling (SEM). At the same time, environmental variables such as annual mean temperature (MAT), annual precipitation (MAP), and drought index (AI) are considered. The lnRR of soil microorganisms includes microbial biomass (fungal and bacterial biomass), diversity (shannon diversity index of fungi and bacteria). and changes in soil pH, SOC, TN, TC due to fertilization, which are related to microbial biomass and diversity. We initiate the SEM procedure by specifying a conceptual model of the hypothesized relationship based on prior theoretical knowledge and validation. In the SEM analysis, we compared the model implied variance-covariance matrix with the observed variance-covariance matrix. Fit the data to the model using the maximum likelihood estimation method. Since some of the variables introduced were not normally distributed, bootstrapping was used to test the probability that the path coefficients differ from zero (Grace, 2006). In order to more accurately identify important indicators affecting microbial biomass and diversity, the least important paths were removed and the model was re-estimated; then the next least important paths were removed, and so on, until retained in the final SEM paths are important. The chi-square test (χ^2) was used to test the overall goodness of the SEM fit. SEM was considered acceptable when the model fit index for χ^2/df was in the range 0.00-2.00 and the p-value was greater than 0.50 (Schermellehengel et al., 2003). We developed a conceptual SEM model with direct effects of environmental variables (MAP, MAT, AI) (latent variables) on microbial biomass (PLFA) and microbial diversity (shannon index) (dependent variables). The amount of N application and duration of the experiment, as well as changes in soil pH (natural logarithm) due to nitrogen fertilization, directly affected microbial biomass and microbial diversity. After completing the conceptual model, we evaluated the conceptual model (full model) versus the reduced model through goodness-of-fit statistics, and used AIC to choose among alternative models. We selected the final model with the lowest AIC value. We implemented SEM using the "piecewiseSEM", “lavaan”, “lme4” package to account for the random effects of "study". All statistical analyses were performed in R 4.1.0.

References

Hedges, L.V., Gurevitch, J. and Curtis, P.S. (1999). The meta - analysis of response ratios in experimental ecology. *Ecology* 80, 1150-1156. doi: 10.1890/0012-9658(1999)080[1150:TMAORR]2.0.CO;2

Dieleman, W.I. and Janssens, I.A. (2011). Can publication bias affect ecological research? A case study on soil respiration under elevated CO_2. *New Phytol.* 190, 517-521. doi: 10.1111/j.1469-8137.2010.03499.x

Dixon, P.M. (2020). The bootstrap and the jackknife: describing the precision of ecological indices. In Design and analysis of ecological experiments pp. 290-318. Chapman and Hall/CRC.

Hedges, L.V. and Olkin, I. (2014). Statistical methods for meta-analysis. Academic press.

Geisseler, D. and Scow, K.M. (2014). Long-term effects of mineral fertilizers on soil microorganisms–A review. *Soil Biol. Biochem.* 75, 54-63. doi: 10.1016/j.soilbio.2014.03.023

Grace, J.B. (2006). Structural equation modeling and natural systems. Cambridge University Press.

Rosenberg, D.M., McCully, P. and Pringle, C.M. (2000). Global-scale environmental effects of hydrological alterations: introduction. *BioScience*, 50, 746-751. doi: 10.1641/0006-3568(2000)050[0746:GSEEOH]2.0.CO;2

Schermelleh-Engel, K., Moosbrugger, H. and Müller, H. (2003). Evaluating the fit of structural equation models: Tests of significance and descriptive goodness-of-fit measures. *Psychol. Methods* 8, 23-74.

Sokal, R.R., Rohlf, F.J. and Rohlf, J.F. (1995). biometry. Macmillan.

Viechtbauer, W. (2010). Conducting meta-analyses in R with the metafor package. *J. Stat. Softw.* 36: 1-48. doi: 10.18637/jss.v036.i03

Supplementary materials

Supplementary Table 1. Most cited references that constituted the intellectual base over three phases

	Rank	five top cited publications (references) in each phase	Local citation score (LCS)	Global citation score (GCS)
The budding period	1	Vitousek PM, et al. 1997. Human alteration of the global nitrogen cycle: sources and consequences. Ecological Applications. 7(3): 737-750	268	3766
	2	Morecroft MD, et al. 1994. An experimental investigation into the effects of atmospheric nitrogen deposition on two semi-natural grasslands. Journal of Ecology. 82(3): 475-483.	67	124

	3	Tilman D, 1993. Species richness of experimental productivity gradients - how important is colonization limitation. Ecology. 74(8): 2179-2191.	47	369
	4	Mounitford JO, et al. 1993. Experimental assessment of the effects of nitrogen addition under hay-cutting and aftermath grazing on the vegetation of meadows on a somerset PEAT moor. Journal of Applied Ecology. 30(2): 321-332.	42	125
	5	Wilson EJ, et al. 1995. Are Calcareous grasslands in the UK under threat from nitrogen deposition - an experimental-determination of a critical load. Journal of Ecology. 83(5): 823-832.	40	65
The fermentation phase (1998-2011)	1	Stevens CJ, et al. 2004. Impact of nitrogen deposition on the species richness of grasslands. Science. 303(5665): 1876-1879.	380	967
	2	Bai YF, et al. 2010. Tradeoffs and thresholds in the effects of nitrogen addition on biodiversity and ecosystem functioning: evidence from inner Mongolia Grasslands. Global Change Biology. 16(1): 358-372.	294	483
	3	Clark CM, et al. 2008. Loss of plant species after chronic low-level nitrogen deposition to prairie grasslands. Nature. 451(7179): 712-715.	280	600
	4	LeBauer DS, et al. 2008. Nitrogen limitation of net primary productivity in terrestrial ecosystems is globally distributed. Ecology. 89(2): 371-379.	250	1476

	5	Bobbink R, et al. 1998. The effects of air-borne nitrogen pollutants on species diversity in natural and semi-natural European vegetation. Journal of Ecology. 86(5): 717-738.	172	893
The "take off" phase (2012-2021)	1	Borer ET, et al. 2014. Herbivores and nutrients control grassland plant diversity via light limitation. Nature. 508(7497): 517.	100	442
	2	Isbell F, et al. 2013. Nutrient enrichment, biodiversity loss, and consequent declines in ecosystem productivity. Proceedings of the National Academy of the United States of America. 110(29): 11911-11916.	98	325
	3	Phoenix GK, et al. 2012. Impacts of atmospheric nitrogen deposition: responses of multiple plant and soil parameters across contrasting ecosystems in long-term field experiments. Global Change Biology. 18(4): 1197-1215.	90	262
	4	Wei CZ, et al. 2013. Nitrogen deposition weakens plant-microbe interactions in grassland ecosystems. Global Change Biology. 19(12): 3688-3697.	73	147
	5	Yang HJ, et al. 2012. Diversity-dependent stability under mowing and nutrient addition: evidence from a 7-year grassland experiment. Ecology Letters. 15(6): 619-626.	59	145

Supplementary Table 2. Top 10 authors, organizations, and countries published publications of nitrogen deposition in grassland. HistCite Pro2.1 is used to analyze and count the number of publications (N), total local citation score (TLCS), and total global citation score (TGCS). VOSviewer is used to count indices of links (L), total link strength (TLS) and citations (C).

Rank	Items	N	TLCS	TGCS	L	TLS	C
1	Han XG	95	1346	4057	84	86	4017
2	Lu XT	61	528	1584	36	34	601
3	Reich PB	53	739	4576	39	30	3175
4	Stevens CJ	50	1439	3689	61	34	2100
5	Wan SQ	47	839	2717	59	35	2316
6	Jiang Y	38	260	769	39	37	758
7	Hobbie SE	36	463	2357	21	24	1776
8	Wang RZ	36	198	542	33	33	501
9	Xu ZW	34	288	898	46	30	841
10	Tilman D	31	1052	7922	17	14	1745

Rank	Items	N	TLCS	TGCS	L	TLS	C
1	Chinese Academy of Sciences	660	4140	18445	184	1126	18344
2	University of Chinese Academy of Sciences	275	1073	4131	111	589	4131
3	University of Minnesota System	134	2027	14196	133	355	13685
4	Lanzhou University	79	324	1456	62	152	1005
5	Colorado State University	72	803	9255	80	153	8649

6	Northeast Normal University China	70	98	445	47	114	446
7	China Agricultural University	69	413	1798	52	132	1798
8	Lancaster University	67	879	3654	95	278	3143
9	Henan University	61	455	1729	44	128	1628
10	Peking University	61	320	2776	60	149	2667

Rank	Items	N	TLCS	TGCS	L	TLS	C
1	China	1053	5025	24616	34	669	63099
2	USA	968	7764	63515	42	809	24616
3	England	338	3307	17874	39	507	17027
4	Germany	246	1382	16704	39	509	17853
5	Australia	197	1054	13332	32	352	13180
6	Switzerland	163	1032	7606	33	330	7458
7	Canada	160	779	9071	31	303	9020
8	Netherlands	146	859	5929	36	284	11930
9	France	112	528	4523	35	277	4508
10	New Zealand	81	187	3141	27	114	8873

Supplementary Table 3. Top 20 journals published studies on nitrogen deposition in grassland from 1990 to 2021.

Journal article	TP	TP(%)	TC	TC/TP	IF

Plant and Soil	127	4.59	3124	24.60	4.19
Global Change Biology	124	4.49	9337	75.30	10.86
Soil Biology & Biochemistry	112	4.05	5171	46.17	7.61
Science of the Total Environment	89	3.22	1560	17.53	7.96
Ecology	75	2.71	9005	120.07	5.50
Oecologia	67	2.42	3185	47.54	3.23
PLos One	66	2.39	1536	23.27	3.24
Environmental Pollution	61	2.21	2315	37.95	8.07
Journal of Ecology	59	2.13	4131	70.02	6.26
Agriculture Ecosystems & Environment	58	2.10	1604	27.66	5.57
Applied Soil Ecology	52	1.88	1071	20.60	4.05
New phytologist	44	1.59	3477	79.02	10.15
Functional Ecology	43	1.56	1506	35.02	5.61
Scientific Reports	42	1.52	786	18.71	4.38
Journal of Vegetation Science	39	1.41	1026	26.31	2.69
Biogeochemistry	38	1.37	1709	44.97	4.83
Ecosystems	38	1.37	1504	39.58	4.22
Plant Ecology	38	1.37	1202	31.63	1.86
Geoderma	33	1.19	771	23.36	6.11
Ecology and Evolution	32	1.16	381	11.91	2.88

Note: TP, number of publications; TP (%), percentage of the journal in the study field; TC, total citation counts; TC/TP, the average number of citations; IF, 2020.
